# Effect of renal denervation on the lipid profile in patients with or without coronary artery disease

**DOI:** 10.1007/s00392-025-02768-4

**Published:** 2025-11-03

**Authors:** Venera Bytyqi, Dennis Kannenkeril, Kristina Striepe, Axel Schmid, Marina V. Karg, Agnes Bosch, Mario Schiffer, Michael Uder, Roland E. Schmieder

**Affiliations:** 1 Department of Nephrology and Hypertension, University Hospital Erlangen, Friedrich-Alexander University Erlangen-Nuremberg (FAU), Ulmenweg 18, 91054 Erlangen, Germany; 2 Institute of Radiology, University Hospital Erlangen, Friedrich-Alexander University, Erlangen, Germany

**Keywords:** Renal denervation, Lipid panel, Coronary artery disease

## Abstract

**Background and aims:**

Sympathetic overactivation plays a critical role in the pathophysiology of various conditions, such as arterial hypertension, chronic kidney disease, coronary artery disease (CAD), diabetes, metabolic syndrome, and dyslipidemia. Initially developed for hypertension management, renal denervation (RDN) has also been associated with metabolic improvements. Preclinical studies in rodent models suggest that RDN may improve lipid profiles by reducing sympathetic activity. This study analyses the effect of RDN on lipid profiles in hypertensive patients with or without CAD.

**Methods:**

This analysis includes 122 hypertensive patients with (*n* = 30) or without CAD (*n* = 92). All patients underwent radiofrequency, ultrasound, or alcohol-injection-based RDN. Fasting lipid profile, including total cholesterol, triglyceride, low-density lipoprotein (LDL), high-density lipoprotein (HDL), and non-HDL levels was measured at baseline and 6 months after RDN in parallel to office and 24-h ambulatory blood pressure (BP).

**Results:**

Six months after RDN, the total cohort showed significant lipid profile improvements. The total cholesterol levels decreased by 10.3 ± 26.3 mg/dL (*p* < 0.001), LDL by 7.0 ± 20.4 mg/dL (*p* < 0.001), and triglycerides by 30.7 ± 69.4 mg/dL (*p* < 0.001), while non-HDL cholesterol levels declined by 7.6 ± 26.3 mg/dL (*p* = 0.002). These changes were independent of BP reduction. In patients with CAD, total cholesterol levels declined by 21.7 ± 29.1 mg/dL (*p* < 0.001), triglycerides by 40.7 ± 80.0 mg/dL (*p* = 0.009), LDL by 15.2 ± 22.0 mg/dL (*p* < 0.001), HDL by 2.8 ± 4.7 mg/dL (*p* = 0.003), and non-HDL by 15.0 ± 34 .8 mg/dL (*p* = 0.021). Reductions in total cholesterol and LDL were greater in CAD than in non-CAD (*p* = 0.011 and *p* = 0.006).

**Conclusion:**

We observed a significant improvement in lipid profiles in hypertensive patients with CAD after RDN. This improvement may represent an additive benefit of RDN in hypertensive patients with CAD.

**Graphical Abstract:**

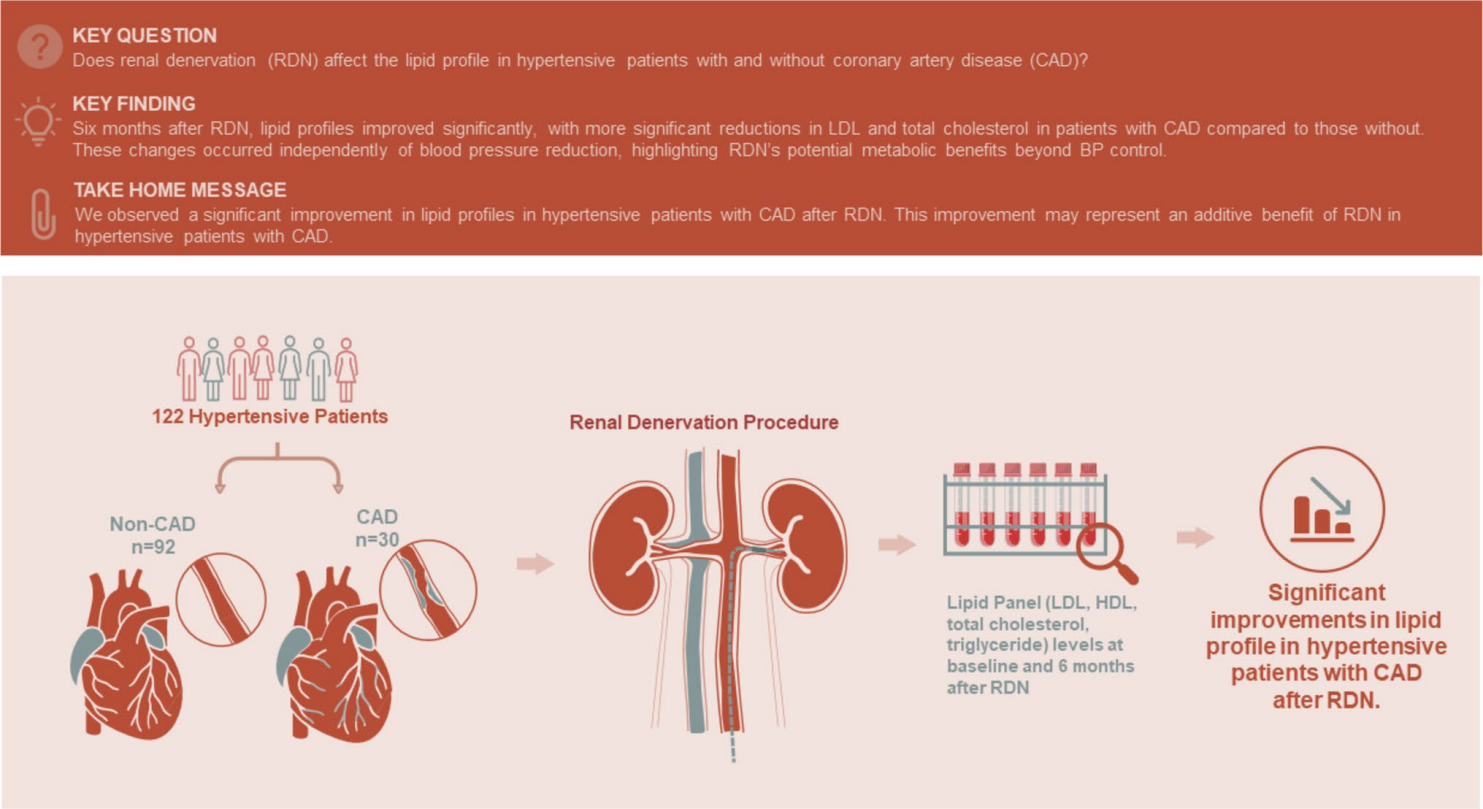

## Introduction

Sympathetic overactivation is a key pathophysiological mechanism underlying a spectrum of cardiovascular and metabolic disorders [1, 2]. While initially recognized for its role in blood pressure (BP) regulation, subsequent research has demonstrated its involvement in chronic kidney disease (CKD), coronary artery disease (CAD), heart failure, diabetes, metabolic syndrome, and dyslipidemia [3, 4]. A deeper understanding of the sympathetic nerve systems’ involvement in these pathophysiological processes has led to targeting sympathetic overactivity as a promising therapeutic strategy to restore physiological balance and improve clinical outcomes [5].

Catheter-based renal denervation (RDN) initially emerged as an innovative therapeutic approach for the treatment of therapy-resistant arterial hypertension. Its underlying mechanism involves the disruption of nerve signaling within the perivascular space surrounding the renal arteries, targeting both afferent sensory and efferent sympathetic fibers, which play a critical role in the development and progression of hypertension and related comorbidities [6–8]. Beyond its established antihypertensive effects, RDN has gained increasing attention for its potential metabolic benefits [5, 9, 10]. Emerging evidence from both clinical and preclinical studies suggests that modulation of renal sympathetic activity through RDN may positively influence glucose homeostasis, enhance insulin sensitivity, and improve lipid metabolism [9, 11, 12]. Of special interest are findings from preclinical studies in rodent models, which indicate that RDN can influence lipid metabolism and potentially improve lipid profiles through mechanisms linked to reduced sympathetic drive [13]. Since hypertension and dyslipidemia often coexist in patients with cardiovascular disease and share common pathophysiological mechanisms, understanding metabolic effects after RDN is clinically relevant.

This study aims to investigate the effects of RDN on lipid profiles in hypertensive patients undergoing RDN, examining changes in key lipid parameters, including total cholesterol, low-density lipoprotein (LDL), high-density lipoprotein (HDL), and triglycerides.

## Methods

### Study design

The Erlangen registry is a single-center, prospective observational registry and involves 122 patients with uncontrolled hypertension who underwent RDN and were prospectively followed up for at least 6 months. Of these, 102 patients were part of the “Renal Denervation in Treatment-Resistant Hypertension” trial (NCT01687725), an investigator-initiated study performed only in our Erlanger center. The remaining 20 patients were involved in various clinical trials, either randomized or non-randomized, sham-controlled or non-sham-controlled studies (NCT02439749), (NCT02649426), (NCT02649426), (NCT02439775), (NCT04311086), (NCT03614260), (NCT04264403). All studies were performed at the Clinical Research Center, Department of Nephrology and Hypertension, University Hospital Erlangen-Nuremberg, Germany. The local Ethics Committee (University of Erlangen-Nuremberg) approved all study protocols, which were conducted in accordance with the Declaration of Helsinki principles. All participants provided written informed consent before study enrollment.

### Study cohort

The study cohort consisted of adult patients with uncontrolled hypertension, including patients with treatment-resistant hypertension on at least three antihypertensive medications (including a diuretic), those taking one to three antihypertensive agents, and medication-naive individuals. Exclusion criteria included secondary arterial hypertension, pregnancy, type 1 diabetes, significant renal artery abnormalities, previous RDN, and contraindications to the RDN procedure (for details, see the above-referenced clin gov numbers and 15–19). In each patient, the diagnosis of uncontrolled hypertension was confirmed through 24-h ambulatory BP monitoring, eliminating potential white-coat hypertension cases. Patients were systematically evaluated to exclude secondary causes of hypertension in accordance with clinical guidelines.

### RDN procedure

The RDN procedure was performed with four different denervation catheters: Radiofrequency-based Symplicity-Flex catheter (Symplicity by Ardian Inc., Palo Alto, CA, USA), radiofrequency-based Symplicity-Spyral catheter (Symplicity by Medtronic, Santa Rosa, CA, USA), ultrasound-based Paradise catheter (Paradise by ReCor Medical, Palo Alto, CA, USA), and alcohol infusion-based Peregrine-system catheter (Peregrine System Infusion Catheter, Ablative Solutions, Inc., Kalamazoo, MI, USA). All interventions were performed via femoral access, with bilateral renal artery treatment completed in a single session. Detailed procedural methodology has been previously described in published literature [14–18].

### Assessments

The baseline assessments included physical examinations, measurements of office and 24-h ABP according to the ESH 2018 guidelines, the collection of medical history and antihypertensive medication data, as well as blood and urine samples. The lipid panel measured the levels of serum total cholesterol, low-density lipoprotein (LDL), triglycerides, high-density lipoprotein (HDL), and non-HDL levels. All measurements were conducted in a fasting state at baseline and repeated at 6 months post-procedure.

### Statistical Analysis

Data analysis was conducted using IBM SPSS Statistics version 29.0 (IBM Corporation, Armonk, NY, USA). The normally distributed variables are presented as mean ± standard deviation in text and tables. Paired samples *t*-tests were used to evaluate changes in lipid parameters (total cholesterol, LDL-cholesterol, HDL-cholesterol, non-HDL-cholesterol, triglycerides) and office and 24-h ABP measurements between baseline and 6-month follow-up. The unpaired t-test was applied to compare continuous variables, and the Chi-square test was performed to compare categorical variables between the groups. Statistical significance was defined as a two-tailed p-value < 0.05. To account for multiple comparisons across lipid parameters, we applied the Benjamini–Hochberg procedure to control the false discovery rate (FDR) at 5%. Statistical significance was defined as a *q*-value < 0.05. Bivariate correlation analyses using Pearson’s test were performed to evaluate the relationship between changes in office-, 24-h ambulatory BP or heart rate (HR) and changes in the lipid panel after RDN.

## Results

### Baseline characteristics

The baseline characteristics of the study population are summarized in Table 1. The cohort consisted of 122 patients diagnosed with uncontrolled hypertension, including resistant hypertension. The participants’ ages ranged from 33 to 79 years, with a mean age of 60 years. The majority of the cohort (70%) were male. Type 2 diabetes mellitus (T2D) was present in 39% of patients, CAD in 25%, dyslipidemia in 43%, and chronic kidney disease (CKD) in 32%.

**Table 1:** Clinical characteristics of the study cohort at the baseline

Clinical characteristics	All (*n* = 122)	CAD (*n* = 30)	Non-CAD (*n* = 92)	*P* value
Demographic data				
Age (years)	60.0 ± 10.4	64.0 ± 9.4	58.8 ± 10.4	**0.014**
Gender (male/female)	85/37	22/8	63/29	0.398
Weight (kg)	90.5 ± 17.0	91.4 ± 17.2	90.2 ± 17.0	0.735
Body mass index (kg/m^2^)	30.1 ± 4.9	31.1 ± 4.8	29.7 ± 4.9	0.168
Comorbidities				
Type 2 diabetes, *n* (%)	47 (39)	18 (60)	29 (32)	**0.005**
Coronary artery disease, *n* (%)	30 (25)	30 (100)	0 (0)	** < 0.001**
Left ventricular hypertrophy, *n* (%)	21 (17)	6 (19)	15 (16)	0.414
Dyslipidaemia, *n* (%)	52 (43)	20 (63)	32 (36)	**0.002**
History of stroke, TIA, *n* (%)	15 (12)	5 (17)	10 (11)	0.292
Chronic kidney disease, *n* (%)	39 (32)	18 (56)	21 (23)	** < 0.001**
Current smoking, *n* (%)	12 (10)	2 (6)	10 (11)	0.658
Office BP				
Office systolic BP (mmHg)	155.7 ± 19.9	153.6 ± 22.7	156.4 ± 19.0	0.516
Office diastolic BP (mmHg)	87.4 ± 13.9	79.3 ± 12.4	90.0 ± 13.5	** < 0.001**
Office heart rate (bpm)	67.8 ± 12.7	68.5 ± 14.6	67.7 ± 12.1	0.780
24-h ABP				
24-h systolic ABP (mmHg)	150.9 ± 14.4	152.6 ± 12.0	150.3 ± 15.1	0.455
24-h diastolic ABP (mmHg)	86.8 ± 11.4	83.2 ± 9.5	88.0 ± 11.8	**0.046**
24-h heart rate (bpm)	66.8 ± 10.1	66.8 ± 11.6	66.8 ± 9.6	0.989
Laboratory values				
HbA1c (%)	6.1 ± 1.0	6.3 ± 0.9	6.0 ± 1.0	0.313
Fasting plasma glucose (mg/dL)	122.3 ± 54.0	137.8 ± 59.2	117.0 ± 51.5	0.069
Serum creatinine (mg/mL)	1.1 ± 0.4	1.3 ± 0.6	1.0 ± 0.3	**0.004**
eGFR (mL/min/1.73 m^2^)	76.8 ± 22.7	65.7 ± 26.0	80.6 ± 20.2	**0.002**
Serum triglyceride (mg/dL)	177.2 ± 98.1	190.8 ± 88.0	172.7 ± 101.2	0.381
Serum total cholesterol (mg/dL)	207.6 ± 47.1	192.4 ± 49.7	212.6 ± 45.4	**0.041**
Serum LDL cholesterol (mg/dL)	139.5 ± 37.7	129.4 ± 40.9	142.8 ± 36.3	0.069
Serum HDL cholesterol (mg/dL)	49.6 ± 14.5	45.4 ± 13.5	51.0 ± 14.6	0.090
Hemoglobin (g/dL)	14.1 ± 1.4	13.6 ± 1.4	14.3 ± 1.4	**0.033**
Hematocrit (%)	41.5 ± 4.1	40.1 ± 4.1	41.9 ± 4.0	**0.033**
Medication				
Number of antihypertensive drugs, *n*	5.0 ± 2.4	6.4 ± 1.8	4.6 ± 2.4	** < 0.001**
Oral antidiabetic, *n* (%)	27 (22)	10 (31)	17 (18)	0.076
Lipid-lowering therapy	43 (35)	19 (63)	24 (26)	** < 0.001**

Data are presented as mean ± SD or *n* (%). *ABP* ambulatory blood pressure, *CKD* chronic kidney disease, *TIA* transient ischemic attack, *LDL* low-density lipoprotein, *HDL* high-density lipoprotein, *HbA1c* glycated hemoglobin, *eGFR* estimated glomerular filtration. Values in bold indicate statistically significant changes (*p* < 0.05)

## Lipid profile in the entire cohort

In the total study cohort, significant alterations in the lipid profile were observed 6 months after renal denervation (RDN) (Table 2). The total cholesterol levels decreased by 10.3 ± 26.3 mg/dL (*p* < 0.001), LDL by 7.0 ± 20.4 mg/dL (*p* < 0.001), and triglycerides by 30.7 ± 69.4 mg/dL (*p* < 0.001). Additionally, HDL levels showed a modest reduction of 1.3 ± 7.2 mg/dL (*p* = 0.054), while non-HDL cholesterol levels declined by 9.0 ± 23.6 mg/dL (*p* < 0.001). The changes in lipid profiles showed no correlation with changes in office and 24-h ambulatory HR, as well as office and 24-h ambulatory BP (*p* > 0.1).

**Table 2:** Changes in lipid profiles at 6 months after RDN

Lipid profile	Baseline	6 months after RDN	*P* value (baseline vs**.** 6 months after RDN)
Total cholesterol (mg/dL)	207.6 ± 47.1	197.3 ± 44.9	** < 0.001**
Triglyceride (mg/dL)	177.2 ± 98.1	146.4 ± 67.8	** < 0.001**
LDL cholesterol (mg/dL)	139.5 ± 37.7	132.5 ± 35.2	** < 0.001**
HDL cholesterol (mg/dL)	49.6 ± 14.5	48.4 ± 14.5	0.054
Non-HDL cholesterol (mg/dL)	158.0 ± 43.2	149.0 ± 40.0	** < 0.001**

Data are presented as mean ± SD. *LDL* low-density lipoprotein, *HDL* high-density lipoprotein, *RDN* renal denervation. Values in bold indicate statistically significant changes (*p* < 0.05)

**Fig. 1:**
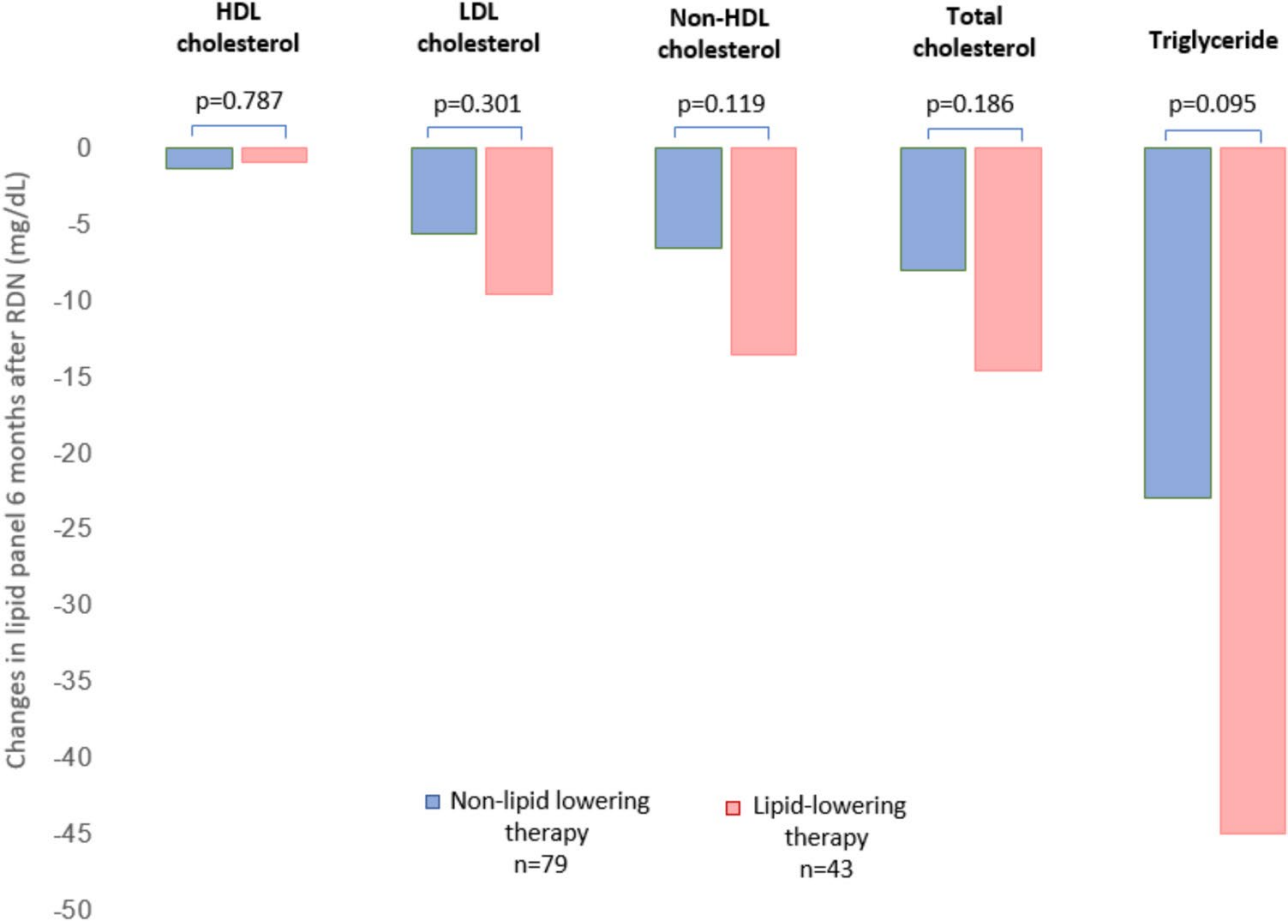
Changes in lipid profiles at 6 months after RDN in patients with and without lipid-lowering therapy

To address potential confounding by concurrent lipid-lowering therapy, a subgroup analysis was conducted, excluding 43 patients receiving lipid-lowering therapy. In this subset (*n* = 79), total cholesterol decreased by 8.0 ± 24.5 mg/dL (*p* = 0.005), LDL by 5.6 ± 19.0 mg/dL (*p* = 0.010), triglycerides by 23.0 ± 61.8 mg/dL (*p* = 0.001), and non-HDL cholesterol by 6.6 ± 21.1 mg/dL (*p* = 0.007) 6 months post-RDN. HDL levels remained unchanged (*p* = 0.131). Furthermore, no significant differences in lipid profiles were observed between patients undergoing lipid-lowering therapy and those not receiving such treatment (see Fig. 1).

### Subgroup analysis: CAD and non-CAD

**Fig. 2:**
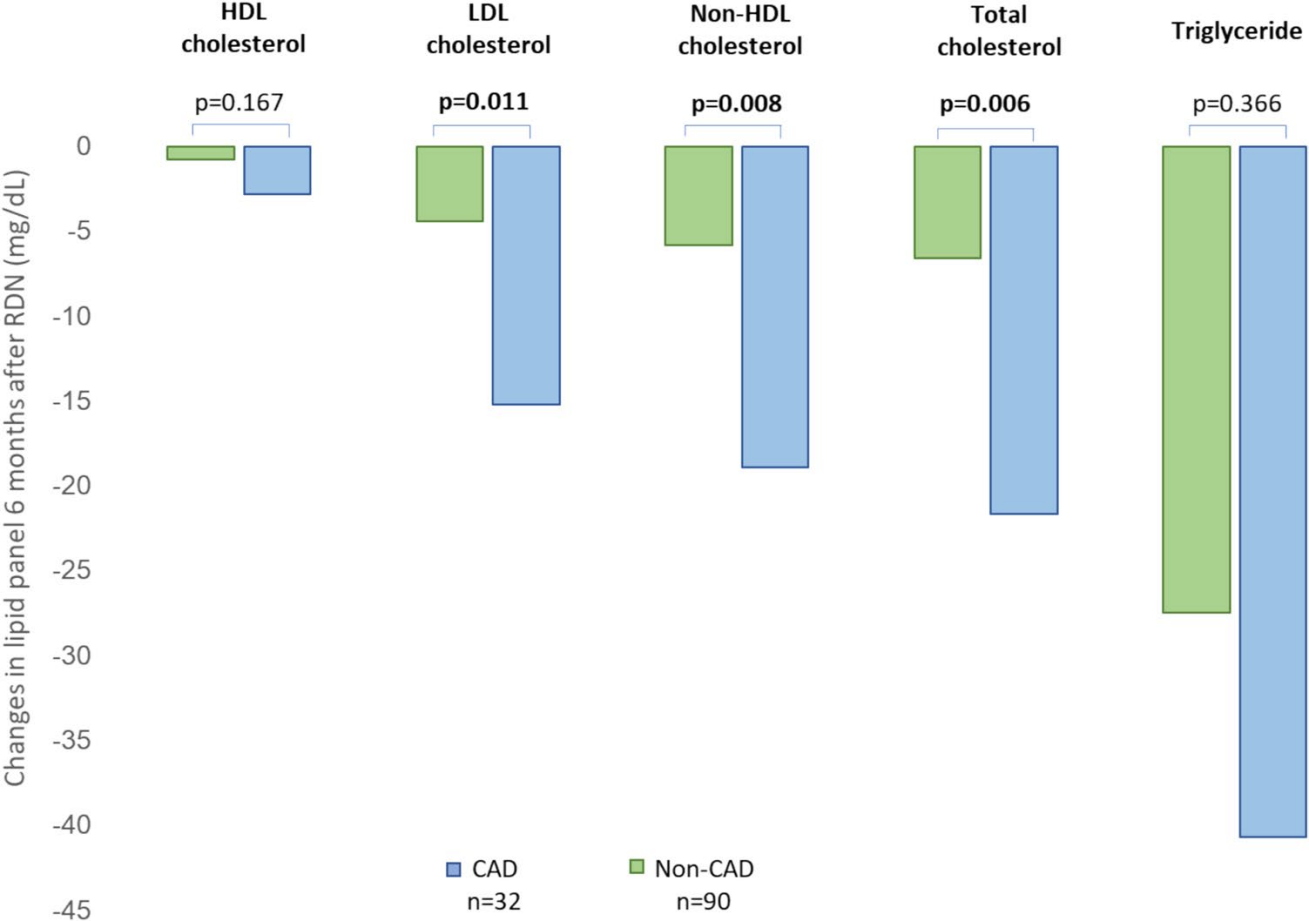
Changes in lipid panel 6 months after RDN in CAD and non-CAD subgroups

A subgroup analysis divided by CAD status was conducted to evaluate lipid changes in this high-risk cardiovascular population. In patients with CAD, mean total cholesterol levels decreased by 21.7 ± 29.1 mg/dL (*p* < 0.001), triglycerides by 40.7 ± 79.5 mg/dL (*p* = 0.009), LDL by 15.2 ± 22.0 mg/dL (*p* < 0.001), HDL by 2.8 ± 4.7 mg/dL (*p* = 0.003) and non-HDL by 18.9 ± 27.3 mg/dL (*p* < 0.001) at 6 months after RDN (Table 3). The reductions in total cholesterol, LDL, and non-HDL levels observed at 6 months post-RDN were greater in the CAD group compared to the non-CAD group (*p* = 0.003, *p* = 0.006, and *p* = 0.008; see Fig. 2).

**Table 3:** Changes in lipid profiles at 6 months after RDN in CAD and non-CAD subgroups B:

Lipid profile	CAD (***n*** = 30)			Non-CAD (***n*** = 92)		
	Baseline	6 months after RDN	*P* value (baseline vs. 6 months after RDN)	Baseline	6 months after RDN	*P* value (baseline vs. 6 months after RDN)
Total cholesterol (mg/dL)	192.4 ± 49.7	170.7 ± 39.9	** < 0.001**	212.6 ± 45.3	206.0 ± 43.1	**0.011**
Triglyceride (mg/dL)	190.8 ± 88.0	150.1 ± 69.9	** < 0.001**	172.7 ± 101.2	145.2 ± 67.5	** < 0.001**
LDL cholesterol (mg/dL)	129.4 ± 40.9	114.2 ± 34.0	**0.009**	142.8 ± 36.3	138.4 ± 33.6	**0.031**
HDL cholesterol (mg/dL)	45.4 ± 13.5	42.6 ± 13.3	**0.003**	51.0 ± 14.6	50.2 ± 14.4	0.355
Non-HDL cholesterol (mg/dL)	147.0 ± 46.9	128.1 ± 37.5	** < 0.001**	161.6 ± 41.6	155.8 ± 38.5	**0.011**

Data are presented as mean ± SD.* LDL*, low-density lipoprotein; *HDL*, high-density lipoprotein; *RDN*, renal denervation. Values in bold indicate statistically significant changes (*p* < 0.05)

### Systolic and diastolic ABP reduction

Across the entire cohort, both office and 24-h ambulatory (BP) demonstrated significant reductions 6 months following RDN, with office BP decreasing by 12.4 ± 19.3/5.7 ± 10.9 mmHg (*p* < 0.001) and 24-h BP by 6.2 ± 14.5/4.1 ± 9.0 mmHg (*p* < 0.001). In the subgroup of patients with CAD, office BP was reduced by 11.1 ± 22.1/4.9 ± 10.3 mmHg (*p* = 0.010, *p* = 0.014), and 24-h BP by 3.9 ± 15.4/3.3 ± 8.3 mmHg (*p* = 0.189, *p* = 0.048), whereas in the non-CAD group, office BP was reduced by 12.9 ± 18.4/5.9 ± 11.2 mmHg (*p* < 0.001), and 24-h BP by 6.9 ± 14.2/4.4 ± 9.3 mmHg (*p* < 0.001), without any significant difference between the two groups.

## Discussion

Our study demonstrates that RDN significantly improves the lipid profiles in hypertensive patients 6 months post-procedure. Previous evidence suggests that modulation of sympathetic nervous system (SNS) activity can beneficially influence metabolic parameters, including lipid metabolism [11, 19]. Pharmacologic inhibition of central sympathetic outflow has been associated with improvements in body weight, glucose regulation, and lipid profiles, suggesting a mechanistic link between sympathetic activity and metabolic homeostasis [5, 19–21].

In our study, we show a significant reduction of total cholesterol, LDL cholesterol, triglycerides, and non-HDL cholesterol levels. Importantly, these improvements persisted even after excluding patients on concurrent lipid-lowering therapy, indicating that RDN effects on lipid metabolism are on top of and independent of pharmacological interventions. Furthermore, we observed no significant difference in the changes in lipid profiles between patients on concurrent lipid-lowering therapy and those not receiving such treatment 6 months after RDN (Table 4).

**Table 4:** Changes in lipid profiles at 6 months after RDN in patients with and without lipid–lowering therapy

Lipid profile	Lipid-lowering therapy (***n*** = 43)			Non-lipid lowering therapy (***n*** = 79)		
	Baseline	6 months after RDN	*P* value (baseline vs. 6 months after RDN)	Baseline	6 months after RDN	*P* value (baseline vs. 6 months after RDN)
Total cholesterol (mg/dL)	190.4 ± 51.1	175.8 ± 43.1	**0.002**	217.0 ± 42.2	209.1 ± 41.6	**0.005**
Triglyceride (mg/dL)	205.4 ± 107.2	160.4 ± 71.5	** < 0.001**	161.8 ± 89.8	138.8 ± 64.9	**0.001**
LDL cholesterol (mg/dL)	125.4 ± 40.8	115.8 ± 35.1	**0.008**	147.2 ± 33.8	141.5 ± 31.9	**0.010**
HDL cholesterol (mg/dL)	46.2 ± 14.3	45.2 ± 14.3	0.190	51.5 ± 14.4	50.1 ± 14.4	0.131
Non-HDL cholesterol (mg/dL)	144.2 ± 47.2	130.6 ± 39.0	**0.002**	165.6 ± 39.2	159.0 ± 37.0	**0.007**

Data are presented as mean ± SD. *LDL* low-density-lipoprotein, *HDL* high-density-lipoprotein, *RDN* renal denervation. Values in bold indicate statistically significant changes (*p* < 0.05)

A subgroup analysis divided by CAD status confirmed these effects in both CAD and non-CAD patients, indicating a consistent response across different cardiovascular risk profiles. The improvement of lipid profile was significantly more pronounced in CAD patients, who showed greater reductions in total cholesterol, LDL-cholesterol, and non-HDL-cholesterol levels compared to non-CAD patients. This observation is remarkable since baseline LDL cholesterol is lower in the CAD group compared to the non-CAD group. According to the law of initial value [22], a lower decrease in lipid levels might be expected, but, in contrast, the changes were more marked in the CAD group, suggesting another potential mechanism. One possible explanation for the greater decrease in lipid profiles in patients with CAD could be the heightened baseline state of SNS overactivity commonly observed in CAD patients [23, 24]. Increased sympathetic tone in this population has been linked to higher muscle sympathetic nerve activity (MSNA), endothelial dysfunction, increased lipolysis, and impaired lipid clearance—all contributing to a more adverse lipid profile [19, 25]. By reducing renal sympathetic outflow, RDN effectively lowers systemic SNS activity, which in turn may improve lipid metabolism. The more pronounced improvements in the CAD group are particularly relevant, as this population typically requires more intensive lipid management as part of secondary prevention strategies [26].

The elevated baseline LDL-C level in the CAD subgroup (mean 129.4 mg/dL) exceeds guideline-recommended targets for secondary prevention. However, this is consistent with real-world data from European registries such as DA VINCI [27] and SANTORINI [28], which report low rates of LDL-C goal achievement despite high statin use. In the German subset of DA VINCI, only 28% of patients achieved the target of < 55 mg/dL. In our study, lipid-lowering therapies were reviewed at baseline and follow-up, and no changes were observed during the study period. This suggests that the lipid improvements observed after RDN were not driven by changes in medication. Nonetheless, the high baseline LDL-C values likely reflect suboptimal background therapy and/or patient nonadherence prior to inclusion, which should be considered when interpreting the effects attributed to RDN.

Interestingly, the reductions in total cholesterol, LDL, and triglycerides observed 6 months after RDN were independent of changes in BP, with no significant correlation between BP reduction and lipid changes. This suggests that the improvements in lipid profiles are unlikely to be a simple downstream consequence of the BP reduction. Instead, these findings support the concept that RDN-induced modulation of sympathetic tone influences lipid metabolism through mechanisms beyond its hemodynamic effects. Evidence shows that the SNS plays a central role in metabolic regulation, influencing hepatic glycogenolysis and gluconeogenesis, adipose tissue lipolysis, skeletal muscle glucose uptake, renal glucose reabsorption, and activation of the renin–angiotensin–aldosterone system (RAAS) [19, 30, 31].

Chronic sympathetic overactivation, as seen in hypertension, contributes to metabolic dysregulation, including disturbances in lipid homeostasis. Experimental studies have shown that the SNS directly promotes hepatic secretion of very low-density lipoprotein triglycerides (VLDL-TG) during fasting [32], further linking sympathetic tone to lipid metabolism. In line with this, Lambert et al. demonstrated an association between dyslipidemia and increased muscle sympathetic nerve activity (MSNA) in young female subjects [33]. Moreover, patients with hypertension and hypercholesterolemia have been shown to exhibit elevated MSNA compared to normotensive individuals [34]. Given that RDN effectively reduces sympathetic tone, it is plausible that the beneficial changes in lipid profiles observed after RDN are mediated through the reduction in SNS-driven metabolic dysregulation.

Additional mechanistic pathways may also underlie the observed findings. Notably, increases in circulating adiponectin have been reported as early as 3 months following RDN in patients with resistant hypertension, independent of concomitant blood pressure changes [35, 36]. Given that adiponectin is secreted by adipose tissue but exerts metabolic effects on hepatic and muscular targets by enhancing fatty acid oxidation and reducing VLDL secretion, this adipose–hepatic axis represents a biologically plausible pathway through which sympathetic modulation could improve lipid metabolism independent of hemodynamic effects.

Furthermore, the observed improvements in lipid profiles following RDN may be partially linked to the activation of the AMP-activated protein kinase (AMPK)/peroxisome proliferator-activated receptor gamma-coactivator 1-alpha (PGC-1α) signaling pathway. Preclinical studies suggest that by reducing sympathetic tone, RDN removes inhibitory signaling, allowing AMPK and PGC-1α to promote lipid oxidation and improve lipid metabolism in diet-induced obese rats. While this molecular link is well-supported in animal models [13, 37], its relevance and the potential for direct metabolic effects in humans remain uncertain and require further investigation. Notably, a systematic review and meta-analysis by Zhang et al. reported only modest improvements in triglycerides and HDL-C after RDN [38], with no significant changes observed in total cholesterol or LDL-C across pooled clinical studies. In the current study, we were able to separately analyze the findings in patients with and without CAD (while controlling for any lipid-modifying therapies), which was not reported in the meta-analysis due to a lack of information reported in the sources. These findings highlight the need for more targeted mechanistic and controlled clinical trials to confirm the metabolic effects of RDN in humans.

## Limitations

While these findings are encouraging, several important limitations should be acknowledged in interpreting the results. This study is a post-hoc analysis of registry data and was not designed as a randomized controlled trial. The lack of a control or comparator group limits our ability to establish a causal relationship between renal denervation and the observed improvements in lipid profiles. Although there were no changes or dose adjustments in lipid-lowering therapy during the follow-up period, we cannot fully exclude the influence of other factors, such as changes in diet, exercise habits, or medication adherence. The renal denervation procedures were performed using different device platforms(radiofrequency and ultrasound). While previous studies have not shown clinically relevant differences in blood pressure outcomes between device types [39], the possibility that these modalities differ in their effects on metabolic parameters, including lipid profiles, cannot be excluded. Objective measurement of adherence through urinary toxicological analysis was conducted only in a subset of patients enrolled in the randomized controlled trial using the second-generation catheter system. To confirm these results and address these limitations, future prospective, double-blind, sham-controlled studies are warranted.

## Conclusion

Our study shows that RDN significantly improves lipid profiles in hypertensive patients, independent of BP changes and pharmacologic lipid-lowering therapy. These findings suggest that RDN exerts metabolic effects beyond its antihypertensive action, potentially through mechanisms linked to sympathetic modulation. The improvement in lipid profiles was particularly pronounced in hypertensive patients with coronary artery CAD. As CAD patients require more aggressive lipid management due to their elevated cardiovascular risk, these findings may offer new insights into the potential metabolic benefits of RDN in this high-risk population.

## Data Availability

Data are available on personal request from the corresponding author.
